# On the Directionality of Cross-Linguistic Effects in Bidialectal Bilingualism

**DOI:** 10.3389/fpsyg.2017.01382

**Published:** 2017-08-15

**Authors:** Tammer Castro, Jason Rothman, Marit Westergaard

**Affiliations:** ^1^Department of Language and Culture, UiT Arctic University of Norway Tromsø, Norway; ^2^School of Psychology and Clinical Language Sciences, University of Reading Reading, United Kingdom; ^3^Department of Language and Literature, NTNU–Norwegian University of Science and Technology Trondheim, Norway

**Keywords:** null objects, Portuguese, bilingualism, bidialectalism, attrition

## Abstract

This study explores the interpretation of null and overt object pronouns by Brazilian Portuguese (BP) and European Portuguese (EP) bidialectal bilinguals. Object pronouns are a particularly good domain to examine, given that, particularly with respect to null objects, the underlying syntax as well as the semantic and discourse constraints that regulate their distributions in the two varieties are superficially different but inherently similar. We test the extent to which native BP speakers who moved to Portugal in adulthood and have lived there for a considerable time display cross-linguistic influence in either direction. Each subject is tested twice, once in BP mode and once in EP mode, which allows us not only to test if they have acquired the EP target structure but also to test the extent to which acquisition of EP might have consequences for the same domain in BP. Our results show that the high degree of typological proximity between the L1 and the L2 may contribute to L1 attrition and hinder target-like performance (i.e., processing) of L2 properties. We relate the findings to key theoretical questions and debates within the context of the larger field of bilingual studies, particularly with respect to L1 attrition and L2 acquisition.

## Introduction

The present study examines attainment in the second language (L2) and retention of the first language (L1) in the same adult native Brazilian Portuguese (henceforth BP) speakers under naturalistic exposure to L2 European Portuguese (henceforth EP). Thus, this study is one of a few in recent years that examines adult L2 acquisition and its potential consequence for L1 maintenance in bidialectalism (see e.g., Cornips, 2014; Garraffa et al., 2015). Even though BP and EP are largely mutually intelligible, under Smith and Wilson (1979, p. 13) conceptualization for determining language status—“[a] language is definable in terms of a set of rules” constituting a unique grammar—there is no question that BP and EP embody distinct grammatical systems. BP and EP present structural differences at all levels (i.e., syntax, semantics, morphology, phonology, discourse, lexis), making it reasonable to consider them distinct languages on linguistic categorizing grounds (e.g., Galves, 2001; Azevedo, 2005). After all, Galician, an equally mutually intelligible Ibero-Romance language, would never be confused as the same language as BP or EP despite the fact that the degree of difference between Galician and BP or EP is not larger than those that distinguish BP from EP (see Fontenla, 2003; Rodrigues, 2004; de Freitas, 2012 for discussion). Although historical reasons conspire to explain why certain genetically related languages of mutual intelligibility are taken to be sub-dialects while others are labeled as fully distinct languages, terminological debates of this type are of little consequence for the present purpose. Whether or not one considers BP and EP to be dialects of a single language or extremely closely related, yet distinct languages, no one familiar with BP and EP would deny that each corresponds to different sets of rules in the Smith and Wilson (1979) sense. For this reason, we consider Brazilians living in Portugal who (seemingly) are speakers of both BP and EP to be (bidialectal) bilinguals, and thus we will refer to BP and EP as languages rather than dialects in this paper. Indeed, examining L2 acquisition and L1 retention in such contexts might be especially illuminating precisely because of the typological relatedness of the languages (see Rowe and Grohmann, 2013; Antoniou et al., 2016; Grohmann et al., 2016 for similar studies on bilectal Cypriot and Standard Greek speakers).

The present study compares and contrasts adult L2 learners—BP natives who moved to Portugal in adulthood—to BP and EP monolingual counterparts. Individuals tested in the L2 target group were primed and tested in both BP and EP in order to trigger different language modes (see Grosjean, 1998, 2008). We capitalize on the null object distribution in BP and EP to test the extent to which: (a) the high degree of typological similarity between the two languages plays a role in the target-like acquisition/processing of L2 structures in EP and (b) the BP used by these Brazilians in Portugal remains unaffected or displays influence from the L2 as a consequence of length of exposure to EP.

In this study, we examine the participants' knowledge of how null objects—phonologically unpronounced, but syntactically present given the verbal argument structure—operate differently across the two languages. Under some analyses, the syntactic status of phonetically unrealized objects in BP and EP is quite distinct (Raposo, 1986), whereas in others the underlying syntactic representations are argued to be very similar (Raposo, 2004), at least sharing some partial overlap. Older analyses that argued for distinctions at the level of syntactic representations had assumed that null objects in syntactic islands are only grammatically possible in BP. This misguided assumption is itself the basis of and the best evidence for claiming differences in BP and EP at the level of underlying syntax. The fact that BP allows null objects in islands must mean that an *in situ* small *pro* is licensed in the grammar. If it were true that the EP grammar precluded null objects in islands as robustly as claimed in earlier work, then it would stand to reason that the syntax of EP and BP must be different. EP presumably does not allow for null objects in islands because the underlying syntax is a topic-operator variable construction (Raposo, 1986), which requires covert extraction (movement) that, by definition, would be blocked by the island itself. The problem, however, is that EP does in fact allow for null objects in islands contrary to Raposo (1986) original intuitions and analysis—as acknowledged in Raposo (2004)—and so it is not at all clear that the underlying syntax of the two languages is different at all. What is clear, however, is that null objects distribute differently in the two languages. Semantic and discourse constraints apply differentially in the two languages, one knock-on effect of which surfaces as a much greater/freer/natural use of null objects in syntactic islands in BP as opposed to EP. Moreover, it had originally been claimed that semantic variables such as animacy only—or most obviously—applied to delimit the distribution (in and outside of islands) of null objects in BP (Schwenter and Silva, 2002). More recent work, however, shows that the very same constraints are also operative—albeit less so—in EP (Duarte and Costa, 2013; Rinke et al., 2016; Castro et al., in revisions). And so, it seems to be the case that null objects can appear in all the same syntactic contexts and are subject to the same semantic and discourse constraints in both BP and EP. However, it is equally clear that in practice null objects are not used in the same ways in the two languages, whereby the constraints that make their use more or less likely have different weightings in BP and EP. We take these differences to be related to processing preferences/strategies as opposed to bonafide grammatical (representation) differences across the languages. Therefore, we will couch the research within this paper as potentially revealing for the interaction interface between bidialectal bilingualism and the application of native target-like processing preferences for the use of null objects. That is, we pursue the idea that crosslinguistic influence, especially in bidialectal bilingualism, can potentially surface as the result of affected processing preferences.

We take advantage of the typological relatedness between BP and EP to test whether the proximity between the L1 and the L2 can contribute to L1 attrition, as has been previously argued (e.g., Altenberg, 1991; Gürel, 2008). Moreover, we make use of these languages' largely shared lexicon to determine whether lexical priming can trigger syntactic co-activation of the L1 (Hartsuiker et al., 2004) or its inhibition, leading to target-like L2 processing (Miller, 2014; Hopp, 2016).

## Typological proximity in the context of L2 processing and L1 attrition

Over the past few decades, first language attrition has been widely discussed in the literature (e.g., Sharwood Smith, 1989; Altenberg, 1991; Köpke, 1999; Cook, 2003; Schmid, 2014). Ecke (2004, p. 322) defines attrition as the “decline of any language (L1 or L2), skill or portion thereof in a healthy individual speaker”. In the case of L1 attrition, speakers who have become highly proficient in the L2 can exhibit signs of cross-linguistic transfer into their native language across various linguistic domains (see e.g., Dussias and Sagarra, 2007; Chang, 2012). The extent to which variation is expected in L1 attriters is attributed to factors such as frequency of L1 usage and length of exposure to the L2. In fact, more interference is expected in speakers who more often use the L1 than in speakers whose L1 is dormant, since both languages are constantly active (Köpke, 2007). Typically, initial stages of L1 attrition are most commonly manifested in word retrieval and processing, especially in near-native speakers of the L2 as a result of a shift in dominance patterns between the two languages (Köpke, 2002; Schmid and Kopke, 2008). Core syntactic computations, however, have been argued to remain unaffected in the L1 of late L2 learners despite prolonged naturalistic exposure to the L2, as L2 interference is commonly found in optionality at the syntax-discourse interface (Tsimpli et al., 2004; Sorace, 2011).

Regarding L1 attrition, Sharwood Smith (1989) has suggested that typological proximity is one of 12 loss-inducing properties (among structural similarity, cross-linguistic support and others). Altenberg (1991), through a case-study of an L1 German couple under naturalistic exposure to L2 English in the United States, also concludes that L1 attrition is more likely to occur when the two languages are typologically similar. Though Altenberg's case-study was based on a small sample size, this conclusion seems to be the consensus among many scholars (see Schmid, 2011, p. 122 for discussion). Gürel (2008) claims that any change in L1 properties can only be triggered by certain L2 forms, as long as they are less complex in the L2 than in the L1, which is generally linked to simplification (see also Seliger, 1989, 1996). On that note, the conclusion is that the integration of the two languages causes L1 and L2 rules to compete, if they are linguistically comparable, which is more likely when the two languages are typologically similar (Köpke, 2007; Paradis, 2007).

Another long-debated issue relates to the facilitative vs. non-facilitative transfer of L1 properties into the L2 (e.g., Flynn and Martohardjono, 1994; Schwartz and Sprouse, 1994, 1996; Lardiere, 1998) and possible L1 interference in L2 processing (e.g., Elston-Güttler et al., 2005; Clahsen and Felser, 2006; Hopp, 2010). With respect to the influence of the lexicon in L2 processing, Hartsuiker et al. (2004) have proposed a shared-syntax model, according to which syntactic co-activation of the L1 can be triggered by its lexical co-activation, as lemma entries appear to be linked to combinatorial nodes of syntactic structures. For instance, when the lemma for the English word *hit* is activated, it consequently activates combinatorial nodes that indicate its grammatical structure—transitive verb, active voice, etc. These combinatorial nodes are then linked to all words in the lexicon, unspecified for language. As a result, L1 syntactic structures could emerge as a result of lexical priming in the L2, provided that they share some syntactic elements, irrespective of typological proximity (see Hartsuiker and Pickering, 2008, for discussion). Naturally, parallel co-activation of the L1 lexicon is more likely to occur when the words are similar in the L2, such as cognate words (Kroll et al., 2013).

It has also been suggested that cognate facilitation can result in greater inhibition of the L1 syntax as a consequence of faster lexical processing in the L2. For example, in a study analyzing cognate vs. non-cognate facilitation for syntactic processing of *wh*-dependencies, Miller (2014) concluded that L1 English-L2 French readers were able to reach target-like syntactic structures more successfully in cases where there was cognate facilitation, and that non-cognate items typically led to errors. Hopp (2016) discusses the results of a study investigating how L2 on-line sentence comprehension can trigger activation of L1 syntax in an L1 German-L2 English population. The results of two eye-tracking tasks indicate that lexical cognate facilitation can help inhibit L1 syntax and thus lead to successful syntactic processing in the L2.

Several studies have been carried out to measure L1 attrition and L1 transfer in late L2 learners, with language pairings that are typologically distant (e.g., Turkish-English in Yağmur, 1997; Greek-English in Pliatsikas and Marinis, 2013) and typologically similar (e.g., Swedish-German in Håkansson et al., 2002; German-English in Hopp, 2016). A high degree of typological relatedness has thus been described as a factor that can contribute to L1 attrition and influence L2 processing. Håkansson et al. (2002) argued that L1 transfer of syntactic properties does not take place in native Swedish learners of L2 German. Bohnacker (2006), however, used the same language pairing to show that transfer from L1 can also occur, in light with the Full Transfer/Full Access Approach (Schwartz and Sprouse, 1994, 1996). The typological relatedness factor is also seen in third language (L3) acquisition, a context in which it should be relatively easy to detect which of the first two languages transfer comes from and whether it is conditioned by how similar they are to the L3. The Typological Primacy Model (Rothman, 2011, 2015) maintains that L3 learners selectively transfer either the L1 or the L2 grammar into the initial stages of L3 acquisition based on the parser's determination of which is typologically closest to the target L3. In a comparative study, Rothman (2011) tested L3 acquisition of BP by a group of L1 Italian-L2 English learners and a group of L1 English-L2 Spanish learners and concluded that transfer emerges from the closest language, regardless of the order in which it was acquired. In this particular case, transfer was from Italian and Spanish, as they are typologically closer to BP than English.

The aforementioned studies are of special relevance to this study, since we provide a language context of two distinct grammars with a mostly shared lexicon, which allows us to investigate the issues raised in this section. Given the high degree of typological relatedness between BP and EP, we have an ideal scenario to test the extent to which L1 attrition and/or L2 processing can be linked to typological relatedness.

## Phonetically unrealized objects

It is well-documented that verbal arguments—subjects and accusative objects—in some of the world's languages can be left phonetically unrealized. Generally speaking, Portuguese is a language that exercises the option to drop accusative arguments. In all Portuguese variants, to our knowledge, accusative arguments can be dropped via VP-ellipsis, a topic operator syntax and/or the licensing of an empty category pronoun under certain conditions.

Although EP and BP are both classified as null object languages, the surface distribution—as alluded to in the introduction section—is quite distinct related to the likelihood of choosing an overt or null object depending on different syntactic environments and semantic features related to object, which give rise to default interpretation preferences notwithstanding the same surface string of words. Before going into the specifics of the differences between BP and EP, it is worth pointing out that both languages only allow 3rd person objects to be null and that all null objects are restricted by the Identification Requirement on empty categories (Rizzi, 1982; see Kato, 1993 for application in these constructions). The Identification Requirement highlights how the syntax for licensing empty categories is a necessary, but not sufficient condition for the production of null arguments, since they must be semantically interpretable. As a result, in order for an argument to be phonetically unrealized it must be in a pragmatic context in which the referent can be recovered by the interlocutor. Apparently, what meets the Identification Requirement in BP and EP is distinct or weighted differently, giving rise to a series of knock-on effects that we recognize as preferences in use and interpretation across the grammars, as described in detail below.

### Brazilian portuguese

Null objects in BP have been described in the literature as an instantiation of the empty category *pro* (Farrell, 1990; Rothman and Iverson, 2013)^1^, since they appear in strong and weak islands alike. Lopes and Santos (2014) point out that both VP-ellipsis and anaphoric null objects can occur in strong islands in BP, as illustrated in (1) and (2), respectively (from Lopes and Santos, 2014, p. 197):

(1)   A: -    O       João    soube    que    você    ia convidar

                  the     João    knew     that    you     were inviting

                  ele pra festa?

                  him to the party?

                  ‘Did John know you were inviting him to the party?’

        B: -    Não,    ele    morreu    antes de    eu    convidar    Ø.

                  no,       he    died    before    I    invited    [-]

                  ‘No, he died before I did it.’

(2)   Ela    comprou    o casaco    quando    experimentou    Ø.

        she    bought      the coat       when       tried                 [-]

        ‘She bought the coat when she tried (it) on.’

The distribution of null and overt objects is not entirely free, however. In order for objects to be dropped in BP, they must be 3rd person, as 1st, and 2nd person referents must remain overt. In addition, pragmatic felicitousness conditions and semantic feature constraints appear to limit their occurrence. Schwenter and Silva (2002) argue that, in order for the object to be null in BP, the referent must be inanimate or non-specific. If the referent is animate and specific, an overt pronoun or DP appears to be obligatory. The specificity constraint is shown in examples (3–4) (from Lopes and Cyrino, 2005, p. 3) and the animacy constraint is illustrated in examples (5–6) (from Schwenter and Silva, 2002, p. 579):

(3)   [+animate,   +specific]

       O policial     insultou o preso antes de torturar

       The policeman insulted._3sg_ the prisoner before of torture._INF_

        *___/ele.

        *___/him

       ‘The policeman insulted the prisoner before torturing (him).’

(4)   [+animate,   −specific]

       O policial     insulta presos antes de torturar

       The policeman insult._3sg_ prisoners before of torture._INF_

       ___/them

       ___/eles.

       ‘The policeman insults prisoners before torturing (them).’

(5)   [−animate, +specific]

       Sabe a árvore grande que tinha na minha rua? A prefeitura

       know-_3sg_ the tree big that had on+the my street? the city

         derrubou **Ø/?ela**.

         hall knocked down she

       ‘You know the big tree that was on my street? City Hall

         knocked (it) down.’

(6)   [+animate, +specific]

       O cachorro da Ana adora ir na rua. Ela sempre

       the dog of+the Ana love._3sg_ go on+the street. she always

          leva ?^*^Ø/ele para passear.

         take-_3sg_ he to walk

       ‘Ana's dog loves to go out in the street. She always takes him

         for walks.’

### European portuguese

Phonetically unrealized objects in EP are also restricted to 3rd person accusative contexts. Early studies argued that the syntax of null objects in EP must be a topic-operator-variable structure as opposed to *pro*, most convincingly argued on the basis of data suggesting that null objects are patently ungrammatical in island contexts. If accurate, having only a topic-operator structure would indeed mean that null objects are precluded from island contexts because they cannot be bound by the topic operator in the matrix clause when necessarily crossing a strong island boundary (Raposo, 1986; Maia, 1997). Data that lead to this conclusion are exemplified in (7) (from Raposo, 1986, p. 382):

(7)  ^*^O    rapaz    que    trouxe Ø agora    mesmo da       pastelaria

        the   boy      who   brought Ø now    just from+the   bakery

        era o teu afilhado.

        was the your godson

        ‘The boy who brought (it) right now from the pastry

        shop was your godson.’

In more recent work, however, Raposo (2004) revises his initial stance, and argues that sentences such as (7) are (marginally) acceptable in EP, and therefore, at least some null objects in EP are instances of *pro* as is the case in BP. According to Raposo (2004), while null arguments within strong islands are not completely ruled out, they are not preferred whereas the null object is highly preferred in simple clause contexts.

It has also been discussed that, unlike what was shown for BP, animacy constraints do not seem to delimit null objects in otherwise possible syntactic environments in EP (Costa and Duarte, 2001; Costa et al., 2009). Since the object referent in (8a) is [−animate, +specific], this sentence is grammatical in both EP and BP; however, (8b) is not possible in BP because it is [+animate] and [+specific], but completely acceptable in EP (from Costa and Duarte, 2001, p. 5):

(8a)  A: E     este  carro?

             and   this   car

             ‘What about this car?’

        B:  O    Zé      quer   saber   quem comprou *ec*.

             the   zé       wants know  who bought *ec*

             ‘Zé  wants  to       know  who bought (it).’

(8b)  A: E     a Maria?

             and  the Maria

             ‘What about Maria?’

        B: O     Zé   quer      saber quem     beijou *ec*.

             the    zé   wants    know              who kissed *ec*

             ‘Zé wants to know                     who kissed (her).’

However, recent work by Duarte and Costa (2013) acknowledges that animacy effects on object drop can also be found in EP in limited contexts. These authors argue that, if the antecedent is within the same sentence, the object can be dropped if inanimate, as shown in (9), but if animate, dropping it is either marginally acceptable or ungrammatical, as illustrated in (10):

(9a) Se achas que *esse livro* é chato, eu não compro Ø para
  if think_2PSG_ that this book is boring_MASC_ I not buy [−] for
  a Maria.
  the Maria
  ‘If you think that this book is boring, I will not buy (it) for
  Maria’.
(9b) Quando encontro *uma gralha*, corrijo Ø imediatamente.
  when find_1PSG_ a typo correct [−] immediately
  ‘When I find a typo, I correct (it) immediately.’
(10a) ??Se achas que *a Maria* é uma chata, eu não convido
  if think_2PSG_ that the Maria is one annoying_FEM_ I not invite
  Ø para a festa.
  [−] to the party
  ‘If you think that Maria is annoying, I will not invite (her)
  to the party.’
(10b) ^*^Quando encontro *o Pedro*, beijo Ø com ternura.
  when meet_1PSG_ the Pedro kiss [−] with tenderness
  ‘When I meet Pedro, I kiss (him) with tenderness.’

In addition, Duarte and Costa (2013) argue that some EP speakers allow for null objects within island contexts, provided that the referent is inanimate, as shown in (11a), but not with animate referents, as can be seen in (11b):

(11a)  A –   E então, *o carro novo*?

                   and so the car new

                   ‘So, what about the new car?’

          B –   A minha mulher está furiosa porque comprei

                   the my woman is furious because bought_1PSG_

                   Ø sem ela saber.

                   [−] without she know_INF_

                   ‘My wife is furious because I bought (it) without her

                   knowing.’

(11b)  A –   E então, *a Maria*?

                   and so the Maria

                   ‘So, what about Maria?’

          B –   ^*^A minha mulher ficou furiosa porque eu beijei

                   the my woman became furious because I kissed_1PSG_

                   Ø na festa.

                   [−] at+the party

                   ‘My wife became furious because I kissed (her)

                   at the party.’

### Some notes about overt pronouns in portuguese

Although this chapter deals with the nature of phonetically unrealized objects in BP and EP, our analysis will focus on how speakers interpret the differences between null and overt object conditions. While it is true that overt objects are the only other default choice—aside from null—, they typically surface differently in the two systems. While EP speakers make use of clitic pronouns in accusative contexts, BP speakers choose strong pronouns, as illustrated in (12) (adapted from Silva, 2015, p. 21):

(12)

- Não empurrei a    Diana. (BP/EP)
  not I-pushed the Diana
  “I did not push Diana.”
- Não empurrei ela. (BP/^*^EP)
  not I-pushed her.
- Não a empurrei. (^*^BP/EP)
  not her-_CL−ACC−3SG_ I-pushed
  “I did not push her.”
- Empurrei-a. (^*^BP/EP)
  I-pushed-her-_CL−ACC−3SG_
  “I pushed-her.”

As a replacement for the overt DP *a Diana* “Diana” in (12a), the strong pronoun *ela* “she” is chosen in BP, as shown in (12b), whereas EP speakers select the clitic *a* “her” instead. These clitics in BP are limited to written formal register and do not surface in colloquial speech (Montrul et al., 2011). EP licenses preverbal clitic placement in certain syntactic environments, as in (12c), but shows a higher preference for postverbal clitics, as in (12d) (see Madeira, 1992; Barrie, 2000 for discussion). These differences may not have a direct impact on the speakers' choice between null and overt pronouns, as this choice is arguably determined by the semantic and syntactic constraints previously discussed. However, they are especially relevant for a better understanding of our experimental design and the discussion of our results.

### Contrastive summary of BP and EP null object distribution

The relevant comparative facts regarding null object distribution in BP and EP are summarized in Table 1:

**Table 1:** Summary of constraints which determine the distribution of null objects in BP and EP.

	**Brazilian Portuguese (BP)**	**European Portuguese (EP)**
Syntactic constraints	Null objects allowed in both strong islands and simple clauses.	Null objects allowed in simple clauses and in some strong islands.
Semantic constraints	Null objects allowed with inanimate referents but ruled out with animate referents, unless non-specific.	Null objects allowed with inanimate referents, but marginally acceptable or ungrammatical with animate referents.

## Hypotheses

Given the similar syntactic nature of the null object in BP and EP, we believe that comparing the performance of the control and target groups in the null conditions exclusively or, alternatively, the overt conditions exclusively would only be valid if the syntax was truly distinct. Thus, any measurable difference in behavior would be shown in an intra-group comparison between null and overt conditions, which indicates the preferences of each group, followed by an inter-group comparison of these preferences. To the extent that there is a link between typological similarity and L1 attrition as has been suggested, whereby the closer the L1 and the L2 are typologically, the more likely the L1 will show signs of the L2 syntax (e.g., Altenberg, 1991; Gürel, 2008; Schmid, 2011 among others), we propose:

(a) The high degree of typological relatedness between BP and EP will lead to measurable L1 attrition. Hypothesis (a) will be confirmed if naturalistic L2 learners of EP display signs of EP-like behavior in their native BP, as measured by their choice of null vs. overt object pronoun. This would be seen under two scenarios: (i) they make no distinction in performance between BP and EP modes and are only different from the BP monolingual controls, or (ii) they do make a distinction between BP and EP but are comparatively different only from the BP controls in such a way that EP effects are noted, for example, an emergent, yet not absolute effect of islandhood.

Taking into account that the syntactic distribution of null objects in the two languages is underlyingly similar, any instantiation of non-monolingual-like behavior by the L2ers should be attributed to difficulties in processing. With respect to possible effects of typological relatedness on L2 processing, we can derive two possible hypotheses:

(b) In light of Hartsuiker et al. (2004), the (extreme) lexical overlap of the L1 and L2 will lead to L1 syntactic co-activation, and consequently, to non-target-like L2 processing as a result of L1 syntactic influence.
(c) Conversely, in light of Miller (2014) and Hopp (2016), the lexical co-activation of the L1 will inhibit the L1 syntactic structure, and as a result, target-like L2 processing will take place.

If our results indicate that L2 learners of EP show influence from BP when in EP-mode, hypothesis (b) will be supported. If, on the other hand, it is not the case that signs of BP structure emerge in their EP, and they reach target-like performance, we will have supportive evidence in favor of hypothesis (c).

## Methodological approach

### Participants

We have already alluded to the three groups participating in the experiment presented in this study. In Table 2 we provide details on their make-up and backgrounds.

**Table 2:** Participant information.

	**L2ers (*n* = 32)**	**BPC–BP controls (*n* = 34)**	**EPC–EP controls (*n* = 32)**
Mean age (at time of testing)	33.1 (range = 22–53)	30.3 (range = 20–54)	27.0 (range = 18–67)
Standard deviation	7.577	7.919	9.708
Mean age of L2 onset	22.9 (range = 13–42)	–	–
Standard deviation	6.700	–	–
Mean length of L2 exposure (years)	10.2 (range = 6–17)	–	–
Standard deviation	3.005	–	–
Mean frequency of BP usage	45.31%	88.97%	21.09%
Mean frequency of EP usage	54.69%	11.03%	78.91%
Standard deviation	15.863	12.417	11.025

The control groups (BPC and EPC) were recruited via social media, networks created by the main author, and collaboration of universities in different regions of Brazil and Portugal. Participants filled out a questionnaire, where they reported their age, age of arrival in Portugal (thus length of EP exposure) and various questions related to a self-assessment of frequency of BP/EP usage. As shown in Table 2, though the target group reported using EP more often than BP, their L1 usage was still quite high, which means that both languages are frequently activated. This frequency was estimated based on their answers to the following question: 'Taking into account the Portuguese language only, what option best describes your linguistic scenario?'. The options were the following, and the values we attributed to them are shown in parentheses.

- I only interact with BP speakers. (BP = 100%, EP = 0%)
- Most of the people I interact with are BP speakers. (BP = 75%, EP = 25%)
- Half of the people I interact with are BP speakers, and the other half, EP speakers. (BP = 50%, EP = 50%)
- Most of the people I interact with are EP speakers. (BP = 25%, EP = 75%)
- I only interact with EP speakers. (BP = 0%, EP = 100%)

The target group was tested in and around the city of Lisbon. All participants had normal or corrected vision and normal hearing.

### Experiment

The experiment was designed to test the subjects' interpretation of null vs. overt accusative objects. There are two versions of the experiment—one in EP and one in BP—which differ in their adjustment with respect to lexis, phonology, and morpho-syntax. The bilingual groups took both versions and the controls took only the version corresponding to their L1. We used an Acceptability Judgment Task (AJT), by which participants judged the acceptability of sentences on a Likert scale of 1–6. Each sentence was preceded by a context to ensure its plausibility.

Experimental items consisted of 80 items—40 items testing the effect of the differences between null and overt object pronouns and 40 fillers which served as target items for another study—and 20 random fillers to ensure equal distribution of acceptable and unacceptable items for both versions of the task. The target items were divided into eight conditions with five items each, as illustrated with examples from each version in (13–20):

(13) null animate in islands (NAI)

(a) BP version
“*- O André convidou a Priscila para um jantar. O que foi*
  que aconteceu?”
“- André invited Priscila to dinner. What happened?”
**“*- O Andr*é *pagou a conta quando Ø levou Ø ao***
  ***restaurante.”***
“- André paid the bill when Ø took Ø to the restaurant.”
(b) EP version
“*- O João convidou a Fernanda para um jantar. O que é*
  que aconteceu?”
“- João invited Fernanda to dinner. What happened?”
**“*- Ele pagou a conta quando Ø levou Ø ao restaurante.”***
“- He paid the bill when Ø took Ø to the restaurant.”

(14) overt animate in islands (OAI)

(a) BP version
“*- O André convidou a Priscila para um jantar. O que foi*
  que aconteceu?”
“- André invited Priscila to dinner. What happened?”
**“*- O Andr*é *pagou a conta quando Ø levou ela ao***
  ***restaurante.”***
“- André paid the bill when Ø took her to the restaurant.”
(b) EP version
“*- O João convidou a Fernanda para um jantar. O que é*
  que aconteceu?”
“- João invited Fernanda to dinner. What happened?”
**“*- Ele pagou a conta quando Ø a levou ao restaurante.”***
“- He paid the bill when Ø took her to the restaurant.”

(15) null inanimate in islands (NII)

(a) BP version
“*- O Guilherme recebeu uma bicicleta da avó. O que foi*
  que aconteceu?”
“- Guilherme got a bike from his grandmother. What
  happened?”
**“*- O Guilherme ficou feliz quando Ø levou Ø pra casa.”***
“- Guilherme was happy when Ø took Ø home.”
(b) EP version
“*- O Tiago recebeu uma bicicleta da avó. O que é que*
  aconteceu?”
“- Tiago got a bike from his grandmother. What
  happened?”
**“*- Ele ficou feliz quando Ø levou Ø para casa.”***
“- He was happy when Ø took Ø home.”

(16) overt inanimate in islands (OII)

(a) BP version
“*- O Guilherme recebeu uma bicicleta da avó. O que foi que*
  aconteceu?”
“- Guilherme got a bike from his grandmother. What
  happened?”
**“*- O Guilherme ficou feliz quando Ø levou ela pra casa.”***
“- Guilherme was happy when Ø took it home.”
(b) EP version
“*- O Tiago recebeu uma bicicleta da avó. O que é que*
  aconteceu?”
“- Tiago got a bike from his grandmother. What
  happened?”
**“*- Ele ficou feliz quando Ø a levou para casa.”***
“- He was happy when Ø took it home.”

(17) null animate in simple clauses (NAS)

(a) BP version
“*- O namorado da Tatiane estava entediado. O que foi que*
  ela decidiu fazer?”
“- Tatiane's boyfriend was bored. What did she decide to
  do?”
**“*- Ø levou Ø pra praia.”***
“- Ø took Ø to the beach.”
EP version
“*- O namorado da Carolina estava entediado. O que é que*
  ela decidiu fazer?”
“- Carolina's boyfriend was bored. What did she decide to
  do?”
**“*- Ø levou Ø para a praia.”***
“- Ø took Ø to the beach.”

(18) overt animate in simple clauses (OAS)

(a) BP version
“*- O namorado da Tatiane estava entediado. O que foi que ela*
  decidiu fazer?”
“- Tatiane's boyfriend was bored. What did she decide to
  do?”
**“*- Ø levou ele pra praia.”***
“- Ø took him to the beach.”
(b) EP version
“*- O namorado da Carolina estava entediado. O que é que ela*
  decidiu fazer?”
“- Carolina's boyfriend was bored. What did she decide to
  do?”
**“*- Ø levou-o para a praia.”***
“- Ø took him to the beach.”

(19) null inanimate in simple clauses (NIS)

(a) BP version
“*- A professora tinha em casa um livro interessante. O que*
  foi que ela fez?”
“- The teacher had at home an interesting book. What did
  she do?”
**“*- Ø levou Ø pra escola.”***
“- Ø took Ø to school.”
(b) EP version
“*- A professora tinha em casa um livro interessante. O que é*
  que ela fez?”
“- The teacher had at home an interesting book. What did
  she do?”
**“*- Ø levou Ø para a escola.”***
“- Ø took Ø to school.”

(20) overt inanimate in simple clauses (OIS)

(a) BP version
“*- A professora tinha em casa um livro interessante. O que*
  foi que ela fez?”
“- The teacher had at home an interesting book. What did
  she do?”
**“*- Ø levou ele pra escola.”***
“- Ø took it to school.”
(b) EP version
“*- A professora tinha em casa um livro interessante. O que é*
  que ela fez?”
“- The teacher had at home an interesting book. What did
  she do?”
**“*- Ø levou-o para a escola.”***
“- Ø took it to school.”

This division was made to include different variables that can be analyzed simultaneously. Hence, we were able to test both animacy and island effects, which are expected to have an impact on the speakers' choice of null vs. overt pronoun in both languages. As can be seen in the sample test items, each version contained the appropriate choice of pronoun for that system—strong pronouns in the BP version and clitic pronouns in the EP version. This was done to ensure that participants would be judging sentences which are natural in spoken language, as the clitic choice is not the preferred option for Brazilians, and the strong pronoun is never selected by EP speakers. This also allowed us to check whether their overt pronoun preferences have undergone cross-linguistic influence, in both directions.

This task was built on an online platform called SurveyGizmo, which allows the user to create and design experiments with pictures, audio, and other media. Each test item was shown on a computer screen with a simultaneous audio recording of the context and the target sentence, in order to enhance the different modes triggered in each testing session (BP-mode vs. EP-mode). A male and a female voice were used for each version, all native speakers of each respective language. The recordings were counterbalanced such that for half the items, a male voice asked the context question and a female voice answered, and vice-versa for the other half. This was done to ensure that the participants were able to distinguish the context from the test sentence and express their preference considering only the latter.

All items, including fillers, were randomized to avoid priming. After reading and listening to the context and the target sentence, the participants were instructed to judge the sentence based on the scale placed immediately below it. Each point on the scale was distinctly labeled to ensure full understanding of their distribution. The scale used in this task is detailed in Table 3.

**Table 3:** Acceptability scale.

	**1**	**2**	**3**	**4**	**5**	**6**
BP	*Péssima*	*Muito Ruim*	*Ruim*	*Boa*	*Muito Boa*	*Excelente*
EP	*Péssima*	*Muito Má*	*Má*	*Boa*	*Muito Boa*	*Excelente*
English	Poor	Very Bad	Bad	Good	Very Good	Excellent

**Figure 1:**
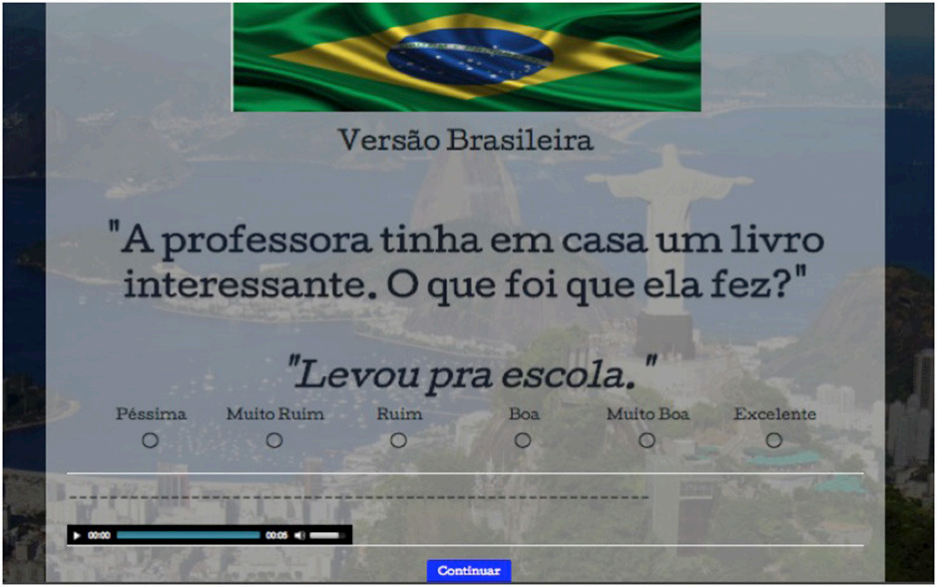
Screenshot, BP version of the task: English: “The teacher had an interesting book at home. What did she do?” “Ø Took Ø to the school” (She took it to the school—NIS condition).

**Figure 2:**
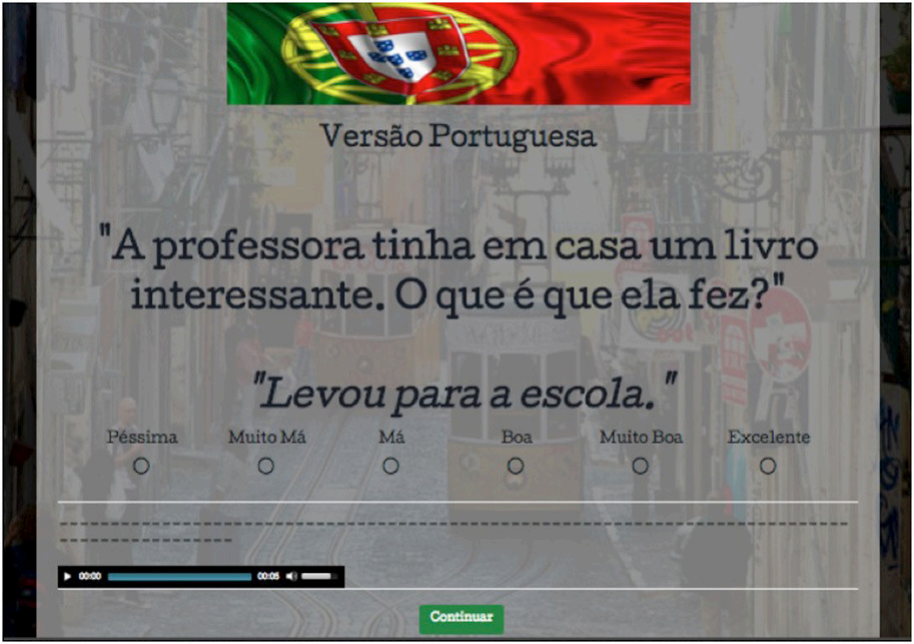
Screenshot, EP version of the task: Same gloss and translation as Figure 1.

Once the participants had made their choice, they clicked on the button *Continuar* (Continue) to move on to the next item. All of their choices were automatically registered online after each click. Figures 1, 2 illustrate screenshots of a random item from the BP and the EP versions respectively:

The semantic and syntactic constraints in the null object distribution patterns in the two languages give rise to potentially different preferences by the target group as a result of interference due to intense exposure to EP, since the monolingual choices are geared by preference rather than grammaticality. This task helps measure the extent to which cross-linguistic influence takes place, and whether it is bidirectional (BP⇔EP), or unidirectional (BP⇒EP or vice-versa).

### Bidialectalism: BP mode vs. EP mode

The most accurate way to check if these speakers are indeed bidialectal bilinguals is to test them separately in BP and EP. Therefore, the L2ers were tested twice, either first in BP, then EP, or the reverse, by native speakers of each variant. Grosjean (1998) has shown that bilinguals display different language modes in their everyday lives (see Grosjean, 1998, 2008 for discussion). In other words, depending on their interlocutor, speakers tend to switch from one language mode to another, even resorting to language mixing such as code-switching and borrowing (Grosjean, 1998). The two versions of the task were very similar, but adjusted for vocabulary distinctions between the two languages. While it is true that the participants all live in Portugal, it may not necessarily be the case that they interact with speakers of both variants to the same extent. Therefore, we included a mode-trigger rapport at the beginning of both sessions. The native EP speaker who conducted the EP version of the tasks started the sessions with general questions about what they liked most about Portugal, e.g., music, films, food, and so on. When tested in BP, they were asked about what they missed from their home country and what sort of connection they still have with Brazil, such as how often they visit, whether they participate in Brazilian events in the area, and so on. After 5 min of chatting, they were considered to be in the mode in which they were about to be tested.

## Results

**Figure 3:**
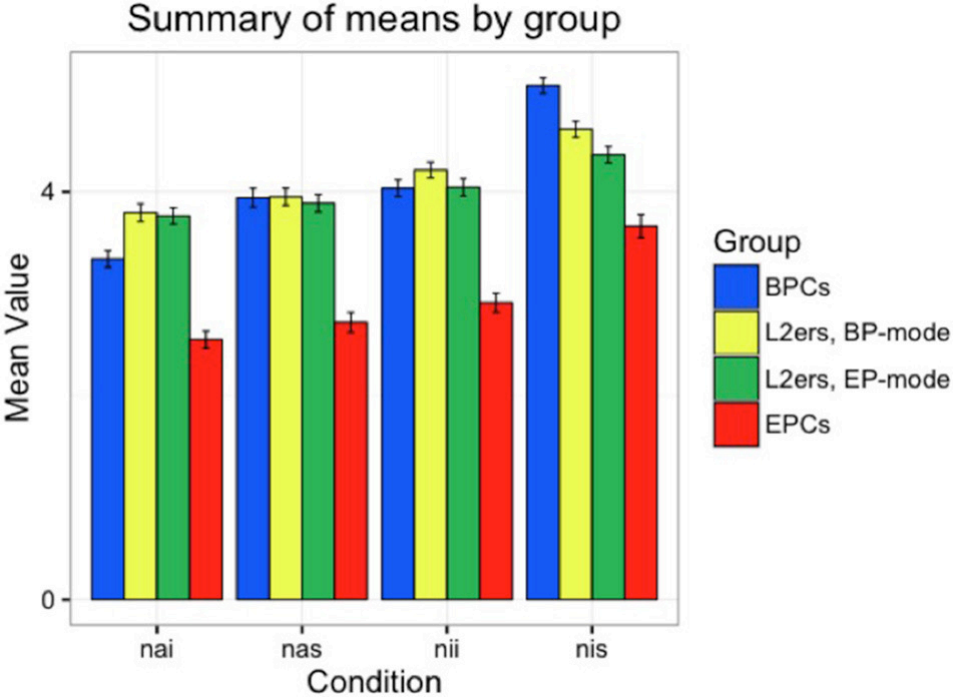
Means by group, null conditions: Visible difference between control groups, though no clear difference shown by target group across the two modes.

**Figure 4:**
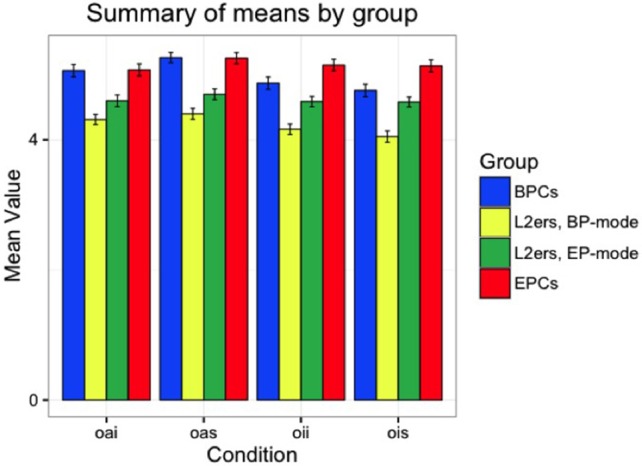
Means by group, overt conditions: No difference between controls, whereas target group shows mode-split.

First, we offer a descriptive analysis of the performance for each group in the null conditions investigated in the Acceptability Judgment Task (AJT), null animates in simple clauses (NAS), null animates in islands (NAI), null inanimates in simple clauses (NIS), and null inanimates in islands (NII), followed by their overt counterparts. Considering 1–6 as the spread of the Likert scale used in the test, judgments above 3.5 were considered “Good,” and below 3.5 “Bad.” Figures 3, 4 show the overall pattern displayed for the null and overt conditions, respectively, across all groups. Table 4 presents the mean values attributed to each condition by group.

**Table 4:** Means by group for each condition, grouped as null conditions (top) and overt conditions (bottom).

**Condition**	**BPCs**	**L2ers–BP-mode**	**L2ers–EP-mode**	**EPCs**
NAI	3.341	3.793	3.762	2.550
NAS	3.941	3.950	3.887	2.718
NII	4.035	4.212	4.043	2.912
NIS	5.041	4.612	4.362	3.662
OAI	5.064	4.312	4.600	5.075
OAS	5.264	4.400	4.700	5.256
OII	4.870	4.162	4.587	5.150
OIS	4.758	4.050	4.581	5.137

We see in Table 4 that BPCs and EPCs differ in their choices, in that null objects are mostly judged as unacceptable by the EP controls and mostly as acceptable by the BP controls. The only exceptions are that EP controls marginally accept them in simple clauses, provided that they have an inanimate referent, and that BP controls marginally judge them below the 3.5 threshold in islands, in contexts where the referent is animate. With respect to the target group, the pattern seen is that the participants do not show differences across the two modes for any of the four null conditions. The results show that L2ers still show BP-like behavior regarding the distribution of null objects, despite over 10 years of exposure to EP.x As for the overt conditions, all groups found the items acceptable (above 3.5), as expected. Note, however, that the L2ers attributed lower values to these sentences than both controls, and showed different behavior across the two modes. Our statistical analysis will help understand which variables have an effect on participants' judgments.

### Statistical analysis

The statistical analysis used for this experiment consisted of mixed-effects models with condition and group as fixed effects. We ran several models in order to consider all variables in our comparisons. First, we look at how the groups interpret the difference between each of the four null conditions and their overt counterparts. We then check for effects of semantic constraints (animates vs. inanimates) and syntactic environment (simple clauses vs. islands). The tables we include in the Appendix (Supplementary Materials) indicate the relevant lines of the models of mixed effects linear regression of all variables analyzed.

**Figure 5:**
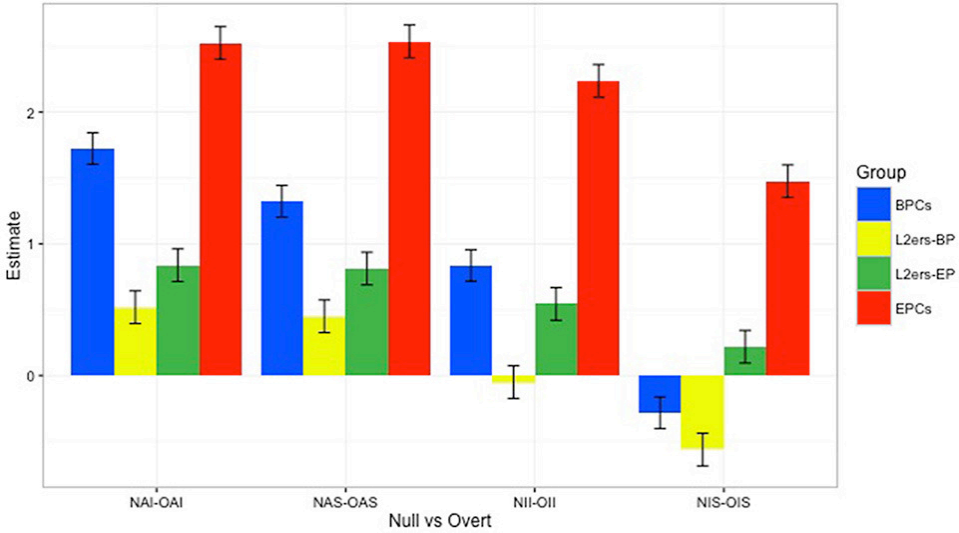
Coefficients of differences between means of null vs. overt conditions across all groups: This illustrates how different the overt conditions are from the null conditions, for each group. The higher the spread, the higher the difference.

BPCs have significantly different values in all four comparisons (see Appendix Table 5 in Supplementary Materials). L2ers do not show a significant distinction between NII and OII in BP-mode (*p* = 0.686), or between NIS and OIS in EP-mode (*p* = 0.077), but all other comparisons show a clear null vs. overt difference for this group in both modes (*p* < 0.05). Like EPCs, BPCs also interpreted all four null conditions to be different from their overt counterparts (and thus assigned different values to them). The spread of the difference between each null and overt context for each of the groups tested is illustrated in Figure 5.

Even though BPCs and EPCs are aware that they should assign different values to sentences with null objects and to sentences with overt objects, the spread of this difference in values is significantly different across the two control groups in all four contexts (*p* < 0.05), (see Appendix Table 6 in Supplementary Materials).

We also found that, in contexts with animate referents within strong islands (NAI and OAI), L2ers interpreted the null vs. overt distinction in BP-mode the same way as they did in EP-mode. However, for the other three environments, they showed statistically different behavior across the two modes, especially with inanimate referents (see Appendix Table 7 in Supplementary Materials). In BP-mode, L2ers only behaved like BP monolinguals regarding the null vs. overt distinction in contexts with inanimate referents in simple clauses (NIS-OIS) (*p* = 0.104). In EP-mode, L2ers patterned with BPCs in contexts with inanimate referents in strong islands (NII-OII) (*p* = 0.091), being significantly different from BP controls in all other comparisons. In both modes, L2ers behaved differently from EPCs with respect to how they interpreted the differences between null and overt in all four environments (see Appendix Table 8 in Supplementary Materials).

Effects of animacy were found for all groups. Participants' assigned values to contexts with null objects are significantly different in items with animate referents than in items with inanimate referents, both in simple clauses and in strong islands (NAI-NII, NAS-NIS), as shown in the Appendix Table 9 in Supplementary Materials. With respect to the syntactic environment, Table 10 in the Supplementary Material illustrates that all groups show a statistical difference between null objects in strong islands and null objects in islands when the referent is inanimate (NIS-NII). BPCs also displayed statistical differences with animate referents (NAS-NAI). This did not seem to be the case for L2ers in either mode and EPCs, for whom the syntactic environment does not affect their judgment on null objects with animate referents.

Lastly, we ran a few additional models (see Appendix Table 11 in Supplementary Materials) focusing solely on the overt conditions, in order to determine whether the morphosyntactic status of overt pronouns—strong pronouns vs. clitic—had a significant effect on the L2ers' preferences. Our results show that L2ers gave significantly lower ratings to sentences with overt pronouns than the monolingual counterparts, in each respective mode (*p* < 0.01).

## Discussion

In this section, we first discuss the results of the interactions between the control groups in light of what current literature predicts. We then discuss the performance of the target group and the comparison to the controls, linking the results to the hypotheses/predictions we made in Section Hypotheses.

### Control groups

The fact that both BPCs and EPCs attributed different values to sentences with null pronouns vs. overt pronouns was not surprising, given that the contexts created took into consideration the variables that arguably determine the null object distribution in each language. The extent to which these two groups interpreted the null vs. overt distinction was not the same, which confirms that the reason why this spread was larger in EP than in BP must be linked to either semantic or syntactic constraints. In light of Schwenter and Silva (2002), we expected BPCs to prefer null objects in contexts with inanimate referents (regardless of syntactic environment). Indeed, we find an effect of animacy for BPCs, as they attributed higher values—though not categorically—to sentences with null objects when the referent was inanimate than to similar sentences with animate referents. EPCs also seemed to show animacy effects, which confirms the arguments made by Duarte and Costa (2013). In fact, when the two control groups are compared to one another, they showed statistically similar behavior with respect to how they interpreted the animacy differences in the contexts given. We conclude from this that the distribution of null objects in BP and EP is not determined, but rather influenced by the [± animate] status of the referent, since the results indicate a general preference rather than a categorical grammatical vs. ungrammatical distinction.

With respect to how the syntactic environment may influence the participants' choices, we expected BPCs to show no effects, as null objects can freely appear within strong islands—provided that the animacy constraints are not violated—, as shown in Rothman and Iverson (2013), Lopes and Santos (2014) and others. Conversely, EPCs are expected to use the syntactic environment as a determining factor for their choice of null vs. overt, in light of Raposo (1986), but in combination with the animacy status of the referent (Raposo, 2004; Duarte and Costa, 2013). Our data show that BPCs display island effects, contrary to what we expected, as the difference in mean values between the contexts with null pronouns (both animate and inanimate) in simple clauses and the same contexts in islands is statistically significant. This suggests that null objects in BP are more likely to occur in contexts with simple clauses than in contexts with strong islands, especially if their referents are inanimate. Therefore, the syntactic environment seems to also have an effect on whether or not the object is likely to be dropped in this language.

EPCs do not make a distinction between these two syntactic environments when the null pronoun has an animate referent—judging both NAS and NAI as equally unacceptable (below the threshold of 3.5)—, but do show a distinction between them when the referent is inanimate, as they judged NIS acceptable but NII unacceptable, as predicted. In other words, the data confirm that null objects in EP are more likely to occur in simple clauses than in strong island contexts, but for inanimate referents only, as the null-object contexts with animate referents were judged unacceptable despite the syntactic environment. When compared to BPCs, the only environment where EPCs show significant differences regarding the syntactic environment was in contexts with null pronouns and animate referents (NAI-NAS), which suggests that the syntactic environment has a stronger effect in EP than in BP. As we pointed out, while the syntactic and semantic constraints appear to be the same in the two systems, the way in which they surface differs, and these differences across the monolingual groups with respect to the syntactic environment serve as evidence for this.

To summarize the comparison between the control groups, we conclude that, even though both the syntactic environment and animacy status appear to have an effect in both BP and EP, the surface distribution of null vs. overt pronominal objects in the control groups is distinct because of the order in which the constraints apply. Our data show that, in EP, the animacy status applies first, and then the syntactic environment, but in BP this order is not clear.

### Target groups

Based on the differences between the control groups with respect to the null vs. overt distinction, we have the tools to discuss the L2ers' performance and the statistical comparisons we made. First of all, L2ers appear to differ significantly from themselves across BP- and EP-modes, except for environments with animate referents in syntactic islands (NAI-OAI). In the other three environments, the null vs. overt distinction for the L2ers was higher in EP-mode than in BP-mode, which reflects the behavior shown by the control groups.

The semantic and syntactic effects also seem to hold for this group. In both modes, L2ers assigned different values to contexts with null objects with animate and with inanimate referents, in both simple clauses and islands. Regarding the syntactic environment, this group also showed significant distinctions in both modes in contexts with null objects and inanimate referents (NII-NIS), but not in similar contexts with animate referents (NAI-NAS). When we consider the null vs. overt distinction, L2ers show significant differences to both control groups in almost all comparisons, except for contexts with inanimate referents in simple clauses (NIS-OIS), where they pattern with BPCs when in BP-mode, and in contexts with inanimate referents in strong islands, where they show BP-like behavior when in EP-mode.

In short, the syntactic environment and animacy status that determine the null object distribution in BP and EP are both at play simultaneously for the target group. Their interpretation of null vs. overt object contexts is highly dependent on these two factors, as it is for monolingual speakers of each language. However, the distinctions between these contexts are mostly interpreted in a way that is different from what was shown by the control groups. Nevertheless, L2ers still behaved BP-like in contexts with inanimate referents in simple clauses (when in BP-mode), and showed signs of the BP distribution when in EP-mode in contexts with inanimate referents in strong islands. No EP-like behavior was detected for these speakers in either mode. Moreover, with respect to overt object pronouns, L2ers gave significant lower ratings than both BPCs and EPCs in each respective mode, which indicates cross-linguistic influence in both directions. Since the clitic option is less preferred in monolingual BP, the lower ratings attributed by L2ers to sentences with overt clitics when in EP-mode can be interpreted as a result of BP=>EP influence. Conversely, sentences with overt strong pronouns were significantly less preferred by the L2ers in BP-mode, which suggests EP=>BP influence. With this in mind, we can link the data to the hypotheses we test in this study.

### Hypothesis (A)–testing for L1 attrition

As discussed in Section Typological Proximity in the Context of L2 Processing and L1 Attrition, recall that several researchers have drawn a link between typological proximity and L1 attrition (e.g., Gürel, 2008; Schmid, 2011). Given the high level of proximity between BP and EP and the length of residence in Portugal of the target group, we hypothesized that these naturalistic L2 learners of EP should display some signs of EP influence in their native BP. As we have shown, the way in which L2ers perceived the differences between contexts with null objects and overt objects when in BP-mode was no longer BP-like, with the exception of one of the contexts (inanimate referents in simple clauses). While not entirely EP-like either, the behavior shown by L2ers in BP-mode indicates that they interpret the null vs. overt distinction differently from monolinguals. As shown in Figure 5, L2ers showed much lower coefficients of differences between the null and overt conditions than the monolingual controls. However, BPCs themselves did not distinguish between the two sets of conditions to the same extent that EPCs did, particularly in the NIS condition. In addition, L2ers in BP mode gave lower ratings to sentences with overt pronouns than BPCs. This can be attributed to the fact that strong pronouns, which are the default choice for most BP monolinguals, are significantly less acceptable to the target group. While it is true that they still judged the sentences as acceptable (well above 3.5), the significant difference between their judgments and the BPCs' judgments suggests a possible effect of EP grammar on their BP. We take this as evidence of cross-linguistic influence from EP to BP instead of a general effect of bilingualism.

### Hypotheses (B) and (C)–testing for L1 effects in L2 acquisition

In light of Hartsuiker et al. (2004), we understand that the lexical co-activation of the L1 should lead to its syntactic co-activation, and as a result, we expected the L2ers in EP-mode to display preferences similar to those of BP monolinguals. Miller (2014) and Hopp (2016), however, defend that the lexical co-activation of the L1 might actually inhibit the L1 syntactic structure, and consequently, L2ers are expected to display target-like performance instead, which would have been manifested if their preferences in EP-mode reflected those of EP monolinguals. We take advantage of the high level of typological proximity between BP and EP to test these two possible outcomes.

Our data show that, in EP-mode, L2ers do not quite reach target-like performance with respect to the EP null vs. overt object distribution, as they are statistically different from EP controls. While also different from BP monolinguals in three of the comparisons, they show BP-like behavior in contexts with inanimate referents in strong islands (NII-OII). We interpret this as an indicator that the BP structure is activated in these speakers' minds, despite the fact that they are in EP-mode. This suggests that, as shown by Hartsuiker et al. (2004), L1 syntax is co-activated with the lexical co-activation of the L1, which was expected given that these two languages share most of the lexicon. Unlike what was shown by Miller (2014) and Hopp (2016), L2ers were not able to fully inhibit their L1 syntax and thus did not reach target-like performance in their L2. Their L1 syntax, despite showing signs of attrition, remains active in the brain, enough to cause them to display some BP-like behavior, even when in EP-mode.

## Conclusion

The conclusions drawn here aim at shedding light on bidialectal bilingualism from a formal linguistic perspective, especially the roles that input and contact play in the acquisition of closely related varieties. In this study, we tested how Brazilians living in Portugal perceive the distribution of null and overt pronominal objects after prolonged exposure to EP, given the apparently different semantic and syntactic constraints that have an effect on their distribution in each language. As it turns out, we find that the animacy status and the semantic environment do not categorically determine the null object distribution in these languages, but rather influence the speakers' preferences. This is because these two factors apply in both BP and EP, but due to strict rule-ordering in the latter, the way in which BP and EP monolinguals deal with the differences between null and overt pronouns is not quite the same. We encourage scholars to consider this conclusion and further explore how null objects are distributed in these two languages.

In this study, we offer some additional evidence in support of the hypothesis that typological proximity is a factor that contributes to L1 attrition (Altenberg, 1991; Gürel, 2008; Schmid, 2011), as our target group, for the most part, no longer patterns with BP monolinguals with respect to the null vs. overt object distribution, displaying cross-linguistic influence potentially stemming from their L2. In addition, we were able to test whether typological proximity hinders or facilitates L2 processing, as compared to what has been shown in previous studies. We conclude from our data that the high degree of similarity between the L1 and the L2 leads to syntactic co-activation of the L1, which results in non-facilitative influence, as previously shown by Hartsuiker et al. (2004).

We strongly believe that the field of L2 acquisition will benefit from further research investigating cross-linguistic transfer, L1 attrition and L2 processing in typologically similar languages, particularly in closely related varieties. In this paper, we have given our small contribution to the field, in the hope that similar studies come to expand on issues raised here.

## Ethics statement

This study was carried out in accordance with the recommendations of Personvernombudet for forskning (ombudsman for research), Norsk samfunnsvitenskapelig datatjeneste AS (Norwegian Social Science Data Services-NSD) with written informed consent from all subjects. All subjects gave written informed consent in accordance with the Declaration of Helsinki. The protocol was approved by the Norsk samfunnsvitenskapelig datatjeneste AS (Norwegian Social Science Data Services-NSD), project number 35815.

## Author contributions

TC is the main author of this manuscript. He has conducted the experiments in person, completed the data analysis and written the majority of the manuscript. JR has given substantial contribution via means of supervision and guidance, and has written some sections of the manuscript. MW has contributed with general and specific comments.

### Conflict of interest statement

The authors declare that the research was conducted in the absence of any commercial or financial relationships that could be construed as a potential conflict of interest.

## Footnotes

^1^There are other proposals suggesting that BP null objects are not instances of *pro*. Kato, for instance, argues that the null object in BP is possibly a case of a null-clitic, because its antecedent has obligatory non-c-commanding status, whereas *pro* can appear both with and without c-commanding antecedents (Kato, 1993). Because the facts of the distribution are best understood by assuming a licensing of *pro*, e.g., subjacency effects, we will follow the *pro* analysis.
