# Supplementary material for: On the Directionality of Cross-Linguistic Effects in Bidialectal Bilingualism
Source: Front Psychol. 2017 Aug 15;8:1382. doi: 10.3389/fpsyg.2017.01382 (PMC5559685; doi:10.3389/fpsyg.2017.01382)
Supplement: Supplementary file 1 [file DataSheet1.DOCX]

Supplementary Material

**On the directionality of cross-linguistic effects in bidialectal bilingualism**

Tammer Castro*. Jason Rothman, Marit Westergaard

*** Correspondence:** Corresponding Author: tammer.castro@uit.no

**Appendix**

For all models run:

Signif. codes: 0 ‘***’ 0.001 ‘**’ 0.01 ‘*’ 0.05 ‘.’ 0.1 ‘ ‘ 1

Residual standard error: 1.108 on 5168 degrees of freedom

Multiple R-squared: 0.2911, Adjusted R-squared: 0.2868

F-statistic: 68.45 on 31 and 5168 DF, p-value: < 2.2e-16

**Table 5. Within-group analysis, with counterpart null conditions as intercept**

| **Intercept BPC** | | | | |
| --- | --- | --- | --- | --- |
| **Variable** | **Estimate** | **Std. Error** | ***t*** | ***p*** |
| conditionOAI | 1.723 | 0.1202 | 14.339 | 0 *** |
| conditionOAS | 1.323 | 0.1202 | 11.011 | 0 *** |
| conditionOII | 0.835 | 0.1202 | 6.949 | 0 *** |
| conditionOIS | 0.282 | 0.1202 | 2.349 | 0.018* |
| **Intercept L2ers (BP-mode)** | | | | |
| **Variable** | **Estimate** | **Std. Error** | ***t*** | ***p*** |
| conditionOAI | 0.518 | 0.1238 | 4.187 | 0 *** |
| conditionOAS | 0.450 | 0.1238 | 3.632 | 0 *** |
| conditionOII | -0.050 | 0.1238 | -0.404 | 0.686 |
| conditionOIS | 0.562 | 0.1238 | 4.540 | 0 *** |
| **Intercept L2ers (EP-mode)** | | | | |
|  | **Estimate** | **Std. Error** | ***t*** | ***p*** |
| conditionOAI | 0.837 | 0.1238 | 6.760 | 0 *** |
| conditionOAS | 0.812 | 0.1238 | 6.558 | 0 *** |
| conditionOII | 0.543 | 0.1238 | 4.389 | 0*** |
| conditionOIS | 0.218 | 0.1238 | -1.766 | 0.077 . |
| **Intercept EPCs** | | | | |
|  | **Estimate** | **Std. Error** | ***t*** | ***p*** |
| conditionOAI | 2.525 | 0.1238 | 20.380 | 0 *** |
| conditionOAS | 2.537 | 0.1238 | 20.481 | 0 *** |
| conditionOII | 2.237 | 0.1238 | 18.060 | 0 *** |
| conditionOIS | -1.475 | 0.1238 | -11.905 | 0 *** |

**Table 6. Comparison between control groups, with counterpart null conditions as intercept**

| **Intercept BPC** | | | | |
| --- | --- | --- | --- | --- |
| **Variable** | **Estimate** | **Std. Error** | ***t*** | ***p*** |
| conditionOIS:groupEPC | -1.757 | 0.1726 | -10.181 | 0 *** |
| conditionOAI:groupEPC | 0.801 | 0.1726 | 4.643 | 0 *** |
| conditionOAS:groupEPC | 1.213 | 0.1726 | 7.033 | 0 *** |
| conditionOII:groupEPC | 1.402 | 0.1726 | 8.123 | 0 *** |

**Table 7. Comparison between L2ers in BP- vs. EP-mode, with counterpart null conditions as intercept**

| **Intercept L2BP** | | | | |
| --- | --- | --- | --- | --- |
| **Variable** | **Estimate** | **Std. Error** | ***t*** | ***p*** |
| conditionOAI:groupL2EP | 0.318 | 0.1752 | 1.819 | 0.068 . |
| conditionOAS:groupL2EP | 0.362 | 0.1752 | 2.069 | 0.038 * |
| conditionOII:groupL2EP | 0.593 | 0.1752 | 3.389 | 0 *** |
| conditionOIS:groupL2EP | -0.781 | 0.1752 | -4.459 | 0 *** |

**Table 8. Comparison between controls vs. L2ers in BP- and EP-mode, with counterpart null conditions as intercept**

| **Intercept BPC** | | | | |
| --- | --- | --- | --- | --- |
| **Variable** | **Estimate** | **Std. Error** | ***t*** | ***p*** |
| conditionOAI:groupL2BP | -1.204 | 0.1726 | -6.979 | 0 *** |
| conditionOAS:groupL2BP | -0.873 | 0.1726 | -5.060 | 0 *** |
| conditionOII:groupL2BP | -0.885 | 0.1726 | -5.129 | 0 *** |
| conditionOIS:groupL2BP | 0.280 | 0.1726 | 1.623 | 0.104 |
| conditionOAI:groupL2EP | -0.886 | 0.1726 | -5.133 | 0 *** |
| conditionOAS:groupL2EP | -0.511 | 0.1726 | -2.960 | 0.003 ** |
| conditionOII:groupL2EP | -0.291 | 0.1726 | -1.689 | 0.091 . |
| conditionOIS:groupL2EP | -0.501 | 0.1726 | -2.903 | 0.003 ** |
| **Intercept EPC** | | | | |
| **Variable** | **Estimate** | **Std. Error** | ***t*** | ***p*** |
| conditionOAI:groupL2BP | 2.006 | 0.1752 | 11.450 | 0 *** |
| conditionOAS:groupL2BP | 2.087 | 0.1752 | 11.914 | 0 *** |
| conditionOII:groupL2BP | 2.287 | 0.1752 | 13.055 | 0 *** |
| conditionOIS:groupL2BP | -2.037 | 0.1752 | -11.629 | 0 *** |
| conditionOAI:groupL2EP | 1.687 | 0.1752 | 9.631 | 0 *** |
| conditionOAS:groupL2EP | 1.725 | 0.1752 | 9.845 | 0 *** |
| conditionOII:groupL2EP | 1.693 | 0.1752 | 9.667 | 0 *** |
| conditionOIS:groupL2EP | 1.256 | 0.1752 | -7.170 | 0 *** |

**Table 9. Within-group analysis, with counterpart animate conditions as intercept**

| **Intercept BPC** | | | | |
| --- | --- | --- | --- | --- |
| **Variable** | **Estimate** | **Std. Error** | ***t*** | ***p*** |
| conditionNII | 0.694 | 0.1202 | 5.775 | 0 *** |
| conditionNIS | 1.100 | 0.1202 | 9.152 | 0 *** |
| conditionOII | -0.194 | 0.1202 | -1.615 | 0.106 |
| conditionOIS | 0.505 | 0.1202 | 4.209 | 0.018* |
| **Intercept L2ers (BP-mode)** | | | | |
| **Variable** | **Estimate** | **Std. Error** | ***t*** | ***p*** |
| conditionNII | 0.418 | 0.1238 | 3.380 | 0 *** |
| conditionNIS | 0.662 | 0.1238 | 5.347 | 0 *** |
| conditionOII | -0.150 | 0.1238 | -1.211 | 0.226 |
| conditionOIS | 0.3500 | 0.1238 | 2.825 | 0.004 ** |
| **Intercept L2ers (EP-mode)** | | | | |
|  | **Estimate** | **Std. Error** | ***t*** | ***p*** |
| conditionNII | 0.281 | 0.1238 | 2.270 | 0.023 * |
| conditionNIS | 0.475 | 0.1238 | 3.834 | 0 *** |
| conditionOII | -0.012 | 0.1238 | -0.101 | 0.919 |
| conditionOIS | 0.118 | 0.1238 | 0.958 | 0.337 |
| **Intercept EPCs** | | | | |
|  | **Estimate** | **Std. Error** | ***t*** | ***p*** |
| conditionNII | 0.362 | 0.1238 | 2.926 | 0.003 ** |
| conditionNIS | 0.943 | 0.1238 | 7.617 | 0 *** |
| conditionOII | 0.075 | 0.1238 | 0.605 | 0.544 |
| conditionOIS | 0.118 | 0.1238 | 0.958 | 0.337 |

**Table 10. Within-group analysis, with counterpart strong-island conditions as intercept**

| **Intercept BPC** | | | | |
| --- | --- | --- | --- | --- |
| **Variable** | **Estimate** | **Std. Error** | ***t*** | ***p*** |
| conditionNAS | 0.600 | 0.1202 | 4.992 | 0 *** |
| conditionNIS | 1.005 | 0.1202 | 8.369 | 0 *** |
| conditionOAS | 0.200 | 0.1202 | 1.664 | 0.096 . |
| conditionOIS | 0.111 | 0.1202 | 0.930 | 0.352 |
| **Intercept L2ers (BP-mode)** | | | | |
| **Variable** | **Estimate** | **Std. Error** | ***t*** | ***p*** |
| conditionNAS | 0.156 | 0.1238 | 1.261 | 0.207 |
| conditionNIS | 0.400 | 0.1238 | 3.229 | 0.001 ** |
| conditionOAS | 0.087 | 0.1238 | 0.706 | 0.480 |
| conditionOIS | 0.112 | 0.1238 | 0.908 | 0.363 |
| **Intercept L2ers (EP-mode)** | | | | |
|  | **Estimate** | **Std. Error** | ***t*** | ***p*** |
| conditionNAS | 0.125 | 0.1238 | 1.009 | 0.313 |
| conditionNIS | 0.318 | 0.1238 | 2.573 | 0.010 * |
| conditionOAS | 0.100 | 0.1238 | 0.807 | 0.419 |
| conditionOIS | 0.006 | 0.1238 | 0.050 | 0.959 |
| **Intercept EPCs** | | | | |
|  | **Estimate** | **Std. Error** | ***t*** | ***p*** |
| conditionNAS | 0.168 | 0.1238 | 1.362 | 0.173 |
| conditionNIS | 0.750 | 0.1238 | 6.054 | 0 *** |
| conditionOAS | 0.181 | 0.1238 | 0.605 | 0.544 |
| conditionOIS | 0.012 | 0.1238 | 0.101 | 0.919 |

**Table 11. Comparison of overt conditions across the language modes**

| **BPC vs. L2ers (BP-mode)** | | | | |
| --- | --- | --- | --- | --- |
| **Variable** | **Estimate** | **Std. Error** | ***t*** | ***p*** |
| conditionOAS | -0.864 | 0.1222 | -7.076 | 0 *** |
| conditionOAI | -0.752 | 0.1222 | -6.156 | 0 *** |
| conditionOIS | -0.708 | 0.1222 | -5.801 | 0 *** |
| conditionOII | -0.708 | 0.1222 | -5.794 | 0 *** |
| **EPC vs. L2ers (EP-mode)** | | | | |
| **Variable** | **Estimate** | **Std. Error** | ***t*** | ***p*** |
| conditionOAS | 0.556 | 0.1240 | 4.485 | 0 *** |
| conditionOAI | 0.475 | 0.1240 | 3.829 | 0 *** |
| conditionOIS | 0.556 | 0.1240 | 4.485 | 0 *** |
| conditionOII | 0.562 | 0.1240 | 4.535 | 0 *** |
